# The use of representative community samples to assess SARS-CoV-2 lineage competition: Alpha outcompetes Beta and wild-type in England from January to March 2021

**DOI:** 10.1099/mgen.0.000887

**Published:** 2023-02-06

**Authors:** Oliver Eales, Andrew J. Page, Sonja N. Tang, Caroline E. Walters, Haowei Wang, David Haw, Alexander J. Trotter, Thanh Le Viet, Ebenezer Foster-Nyarko, Sophie Prosolek, Christina Atchison, Deborah Ashby, Graham Cooke, Wendy Barclay, Christl A. Donnelly, Justin O’Grady, Erik Volz, The COVID-19 Genomics UK (COG-UK) Consortium†, Ara Darzi, Helen Ward, Paul Elliott, Steven Riley

**Affiliations:** ^1^​ School of Public Health, Imperial College London, London, UK; ^2^​ MRC Centre for Global infectious Disease Analysis and Abdul Latif Jameel Institute for Disease and Emergency Analytics, Imperial College London, London, UK; ^3^​ Quadram Institute, Norwich, UK; ^4^​ Department of Infectious Disease, Imperial College London, London, UK; ^5^​ Imperial College Healthcare NHS Trust, London, UK; ^6^​ National Institute for Health Research Imperial Biomedical Research Centre, London, UK; ^7^​ Department of Statistics, University of Oxford, Oxford, UK; ^8^​ COVID-19 Genomics UK Consortium, UK; ^9^​ Institute of Global Health Innovation at Imperial College London, London, UK; ^10^​ MRC Centre for Environment and Health, School of Public Health, Imperial College London, London, UK; ^11^​ Health Data Research (HDR) UK London at Imperial College, London, UK; ^12^​ UK Dementia Research Institute at Imperial College, London, UK

## Abstract

Genomic surveillance for SARS-CoV-2 lineages informs our understanding of possible future changes in transmissibility and vaccine efficacy and will be a high priority for public health for the foreseeable future. However, small changes in the frequency of one lineage over another are often difficult to interpret because surveillance samples are obtained using a variety of methods all of which are known to contain biases. As a case study, using an approach which is largely free of biases, we here describe lineage dynamics and phylogenetic relationships of the Alpha and Beta variant in England during the first 3 months of 2021 using sequences obtained from a random community sample who provided a throat and nose swab for rt-PCR as part of the REal-time Assessment of Community Transmission-1 (REACT-1) study. Overall, diversity decreased during the first quarter of 2021, with the Alpha variant (first identified in Kent) becoming predominant, driven by a reproduction number 0.3 higher than for the prior wild-type. During January, positive samples were more likely to be Alpha in those aged 18 to 54 years old. Although individuals infected with the Alpha variant were no more likely to report one or more classic COVID-19 symptoms compared to those infected with wild-type, they were more likely to be antibody-positive 6 weeks after infection. Further, viral load was higher in those infected with the Alpha variant as measured by cycle threshold (Ct) values. The presence of infections with non-imported Beta variant (first identified in South Africa) during January, but not during February or March, suggests initial establishment in the community followed by fade-out. However, this occurred during a period of stringent social distancing. These results highlight how sequence data from representative community surveys such as REACT-1 can augment routine genomic surveillance during periods of lineage diversity.

## Data Summary

All supporting data, code and protocols have been provided within the article or through supplementary data files, including COGUK_Authors and Accession Numbers.

**Impact Statement**

Genomic surveillance of SARS-CoV-2 has been crucial in detecting new variants, which are more transmissible or better able to evade population immunity. However, standard surveillance relies on a variety of sampling methods, many of which can lead to biassed analysis of the competition between variants. We present analysis of sequences obtained via random sampling, a relatively unbiased sampling method. Sequences were obtained from the REACT-1 study, one of the few (we know of two) infection prevalence studies that ran for a significant period of time during the SARS-CoV-2 pandemic. Sequences were obtained from January 2021 to March 2021 and thus represent some of the earliest sequences obtained via random sampling. Our findings that Alpha outcompeted Beta during this period differed from what was observed in publicly available sequencing data, obtained from community testing; this highlights how random sampling studies can augment standard surveillance methods.

## Introduction

Since the emergence of SARS-CoV-2 in late 2019 [1] there has been a continuous accumulation of mutations leading to a genetically diverse phylogeny [2]. Although most mutations are neutral, having no effect on the epidemiology of the virus, some have been found to affect transmissibility [3] and antigenicity [4], and have arisen on multiple occasions in independent lineages [5]. Lineages that are judged likely to have increased transmissibility or severity relative to current dominant lineages, leading to a change in the epidemiology of the virus, are termed ‘Variants of Concern’ (VOC) [6–8].

The Alpha VOC (B.1.1.7 in pango nomenclature [9]) was first detected in Kent, England on 20 September 2020 [10]. After its emergence, it rose to become the dominant lineage in the United Kingdom (UK), and increased in frequency in many other countries [11] before eventually being outcompeted by the Delta variant [12]. Previous studies have estimated that this lineage was more transmissible than the previously dominant wild-type lineages, as measured by the reproduction number (R) [8, 13].

The Beta VOC (B.1.351 in pango nomenclature) was first detected in South Africa [7] in October 2020 and by March 2021 there had been 291 detections in the UK [14]. The lineage is associated with the E484K SNP in the spike protein, which has been found to reduce the neutralizing activity of post-vaccination sera [15], triggering fears of lowered vaccine efficacy. This SNP was also detected in a cluster of Alpha cases in England, predominantly in the South West (by February 2021) [16]. Two further lineages, A.23.1 [17] and the Eta variant (B.1.525 in pango nomenclature), were also described as ‘Variants under Investigation (VUI)’ in the UK [18] due to the presence of several SNPs of biological significance. Both of these lineages had been detected in low numbers in the United Kingdom [19, 20] during early 2021. A cluster of A.23.1 that exhibits the E484K SNP was detected in Liverpool, England 10 January 2021 [16].

Extensive genomic surveillance has been undertaken in the UK by the COVID-19 Genomics UK Consortium (COG-UK) [21]. From its inception in March 2020 to the end of March 2021, COG-UK sequenced over 430 000 positive cases [22] representing 45% [23] of all uploaded sequences to GISAID, a global open-access database for coronavirus and influenza genomic data [24], with UK coverage of all detected samples varying from 2.5% [25] to 56.2% [22]. Samples included in COG-UK data are taken from several different sources: hospital cases, routine community surveillance, outbreak investigations, and border screening. Potential biases present in these sampling strategies can lead to samples that are unrepresentative of the population at large.

The REal-time Assessment of Community Transmission-1 (REACT-1) study obtains throat and nose swabs from a random sample of the population in England [12]. Due to the random nature of its sampling procedure, it is relatively unbiased compared to other surveillance strategies. From the beginning of May 2020 to the end of March 2021, there were ten rounds of REACT-1 with between 140 000 and 175 000 swab tests each round [12]. Here we present the results of the genome sequencing performed on the positive swabs in early 2021 (genomic sequencing not performed for rounds in 2020), from round 8 (6–22 January), round 9 (4–23 February) and round 10 (11−30 March), a period over which Alpha reached fixation.

**Fig. 1.**
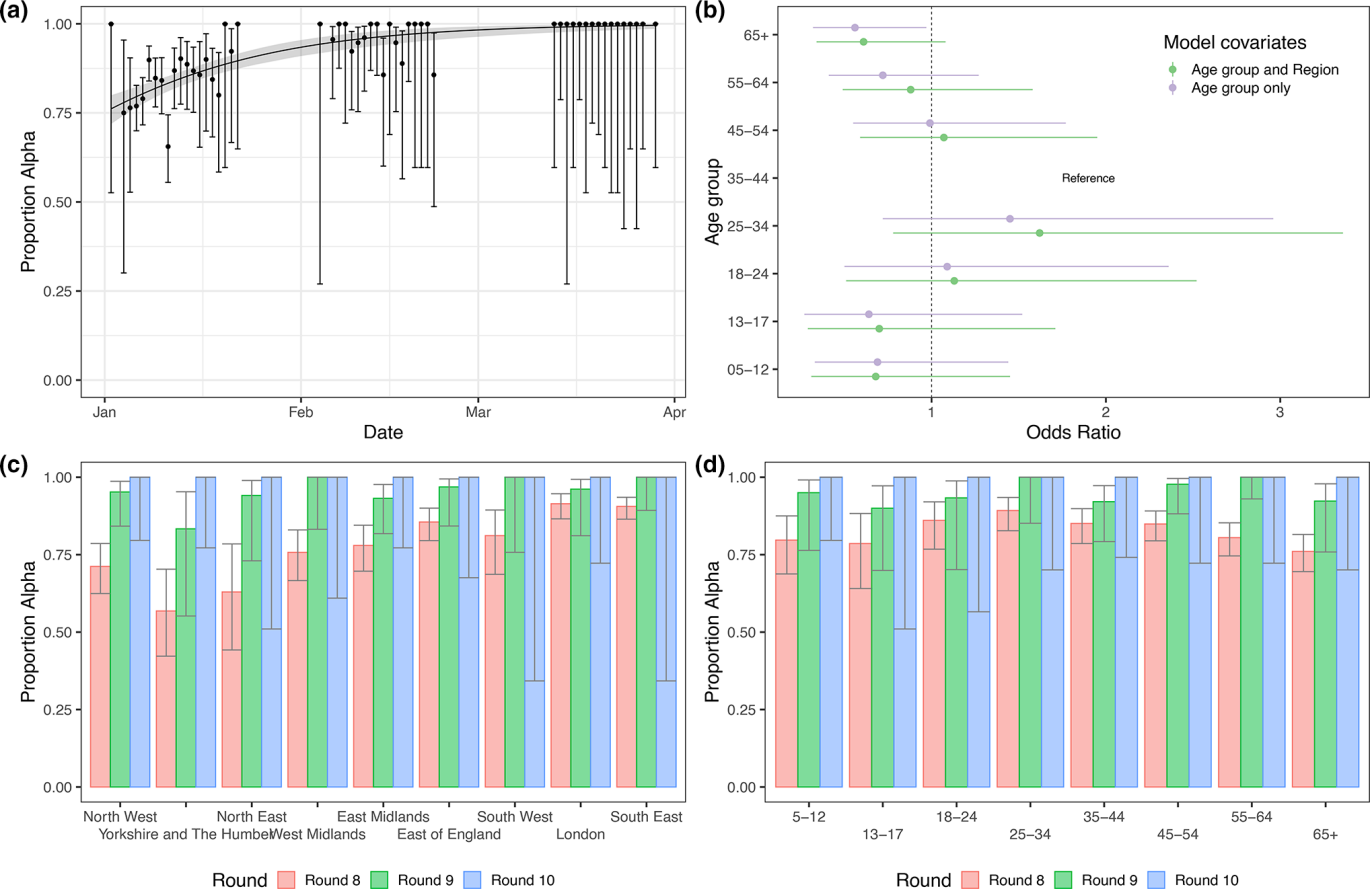
The Alpha variant in England from January to March 2021. (a) Proportion of the Alpha variant over time. Points show raw data with error bars representing the 95% confidence interval. Shaded region shows the best fit Bayesian logistic regression model with 95% credible interval. (b) Odds ratio of a determined lineage being Alpha by age group for logistic models including just age group (purple) and both age group and region (green) fit to data from round 8 only. (c) Proportion of positive tests that are from the Alpha variant by region of England. Error bars show the 95% confidence intervals. (d) Proportion of positive tests that are from the Alpha variant by age group. Error bars show the 95% confidence intervals.

**Fig. 2.**
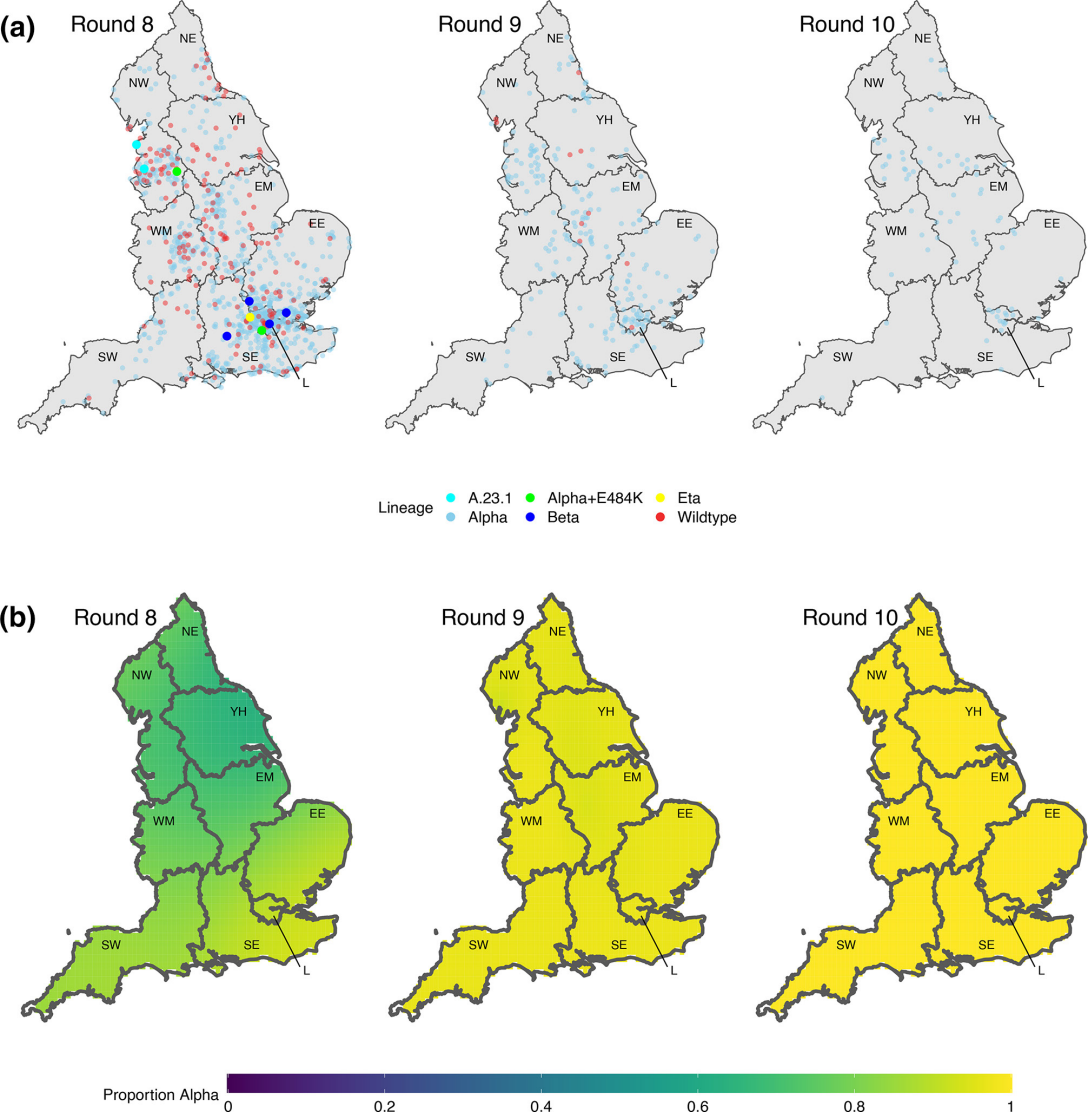
Geospatial patterns of lineage frequency. (a) Location of all positive samples for which we have identified their lineage for each round (each point moved randomly a small distance). (b) Modelled proportion of the Alpha variant across space for round 8, round 9, and round 10. Regions: NE = North East, NW = North West, YH = Yorkshire and The Humber, EM = East Midlands, WM = West Midlands, EE = East of England, L = London, SE = South East, SW =South West.

**Fig. 3.**
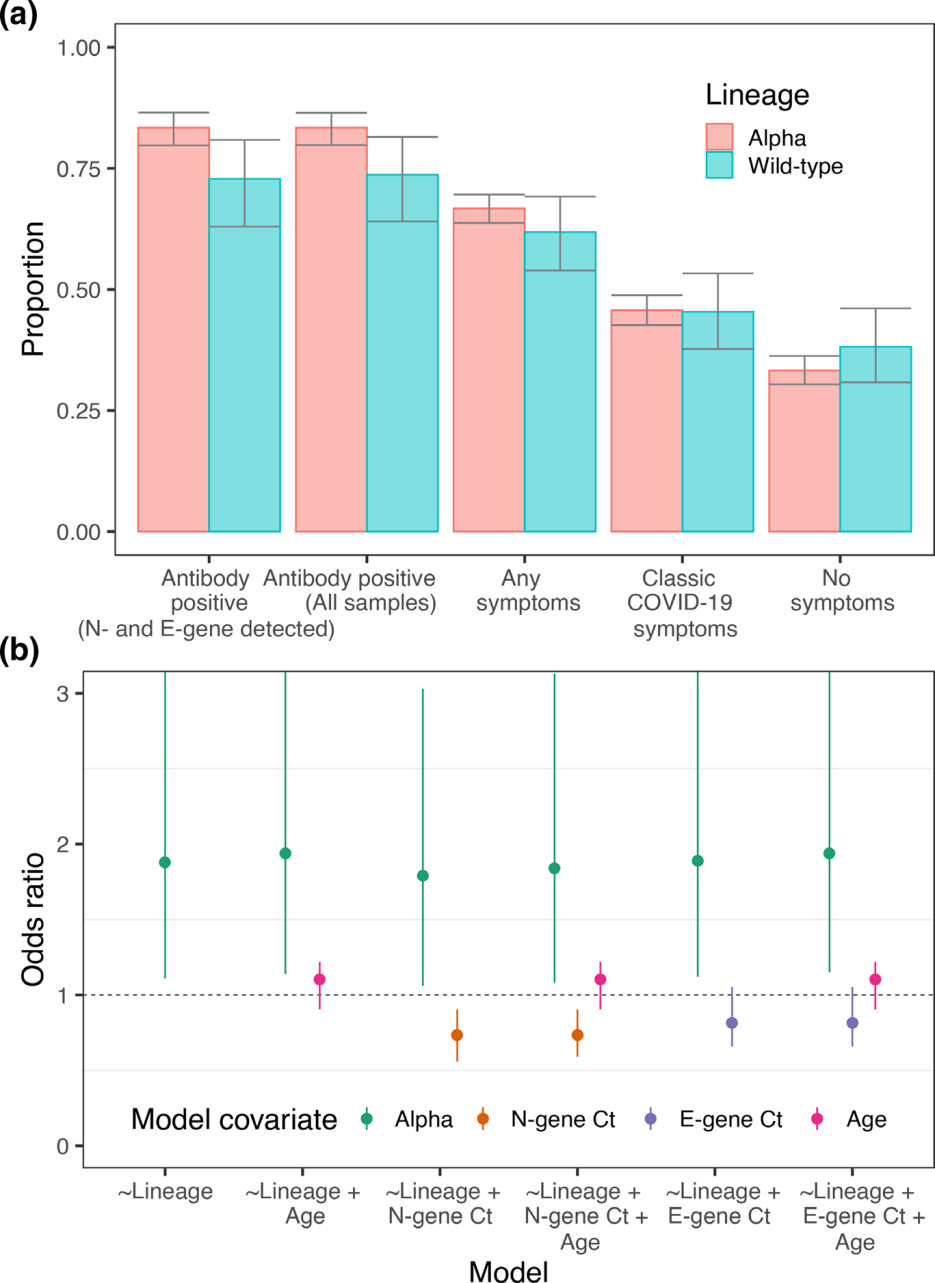
Symptoms and antibody positivity. (a) Proportion of those infected testing positive for antibodies 6 weeks after swab test (for all samples and for those that had both N- and E-gene detected), displaying any symptoms in the week prior to their swab test, displaying classic COVID-19 symptoms (loss of sense of taste, loss of sense of smell, new persistent cough, fever) in the week prior to their test and displaying no symptoms. (b) Odds ratios of the covariates of multiple logistic regression models. Each model had the result of the LFIA antibody test as the outcome variable with different combinations of lineage, N-gene Ct, E-gene Ct and age as the covariates. OR displayed for Alpha is relative to wild-type. OR displayed for N- and E-gene Ct is relative to a change in Ct of +5. OR displayed for age is relative to a change of +10 years in age.

**Table 1.** Results of Gaussian regression with either E-gene or N-gene Ct value as the 619 observation and lineage as the explanatory variable

N- and E-gene positive						N-gene positive		
		N-gene		E-gene			N-gene	
Lineage	Number	Ct value	*P* value	Ct value	*P* value	Number	Ct value	*P* value
Wild-type	182	23.97 (23.29, 24.64)	ref	24.72 (24.01, 25.43)	ref	191	24.41 (23.71, 25.11)	ref
Alpha	1157	22.64 (22.37, 22.90)	0.0003	23.81 (23.53, 24.10)	0.0203	1197	22.97 (22.69, 23.24)	0.0002
Beta	4	26.60 (22.04, 31.15)	0.2640	27.12 (22.34, 31.90)	0.3298	4	26.60 (21.77, 31.42)	0.3791
Eta	1	24.70 (15.59, 33.82)	0.8752	25.97 (16.42, 35.53)	0.7973	1	24.70 (15.05, 34.35)	0.9526
A.23.1	2	24.96 (18.51, 31.40)	0.7650	26.14 (19.39, 32.90)	0.6806	2	24.96 (18.14, 31.78)	0.8752
Alpha+E484K	2	17.45 (11.01, 23.90)	0.0489	19.38 (12.63, 26.14)	0.1239	2	17.45 (10.63, 24.27)	0.0470

Regression models were run on the subset of data that had detected both the E- and N-gene, and the entire dataset, for which N-gene was detected every time.

**Fig. 4.**
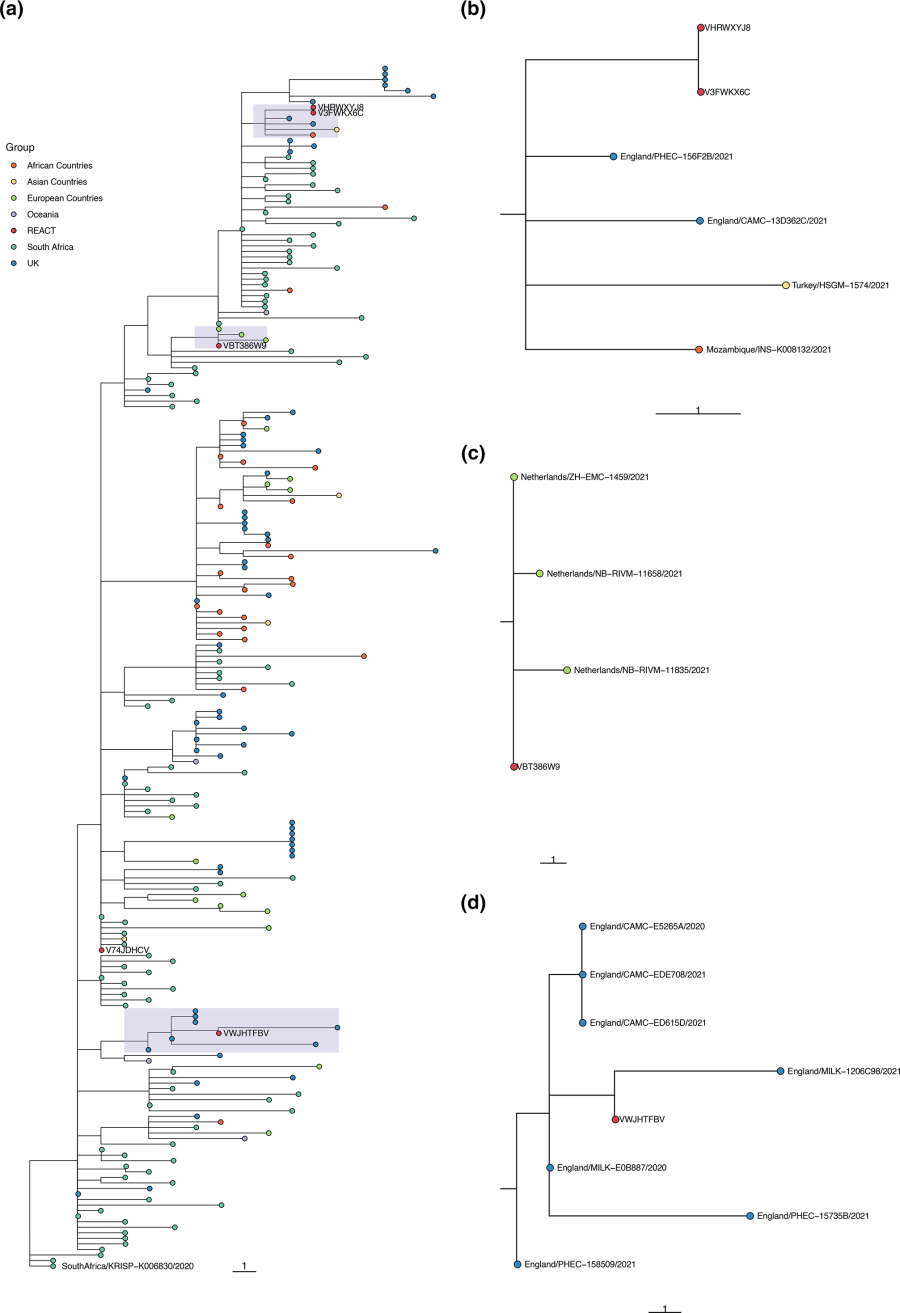
Phylogenetic tree showing the relation of Beta lineages detected in REACT-1 to other Beta sequences in the COG-UK database. Sequences are coloured by the location in which the sequence was isolated. REACT lineages are coloured red and have an ID beginning with the sequence ‘ARCH-”- next to them. (a) The subgroup of the entire constructed tree that contains all REACT sequences, re-rooted to the COG-UK sequence SouthAfrica/KRISP−K006830/2020. (b–d) Zoomed-in view of the subtrees shown by the three shaded regions. Note that adjacent sequences ARCH-000047A3 and ARCH-000052A7 are multiple readings from the same individual.

**Fig. 5.**
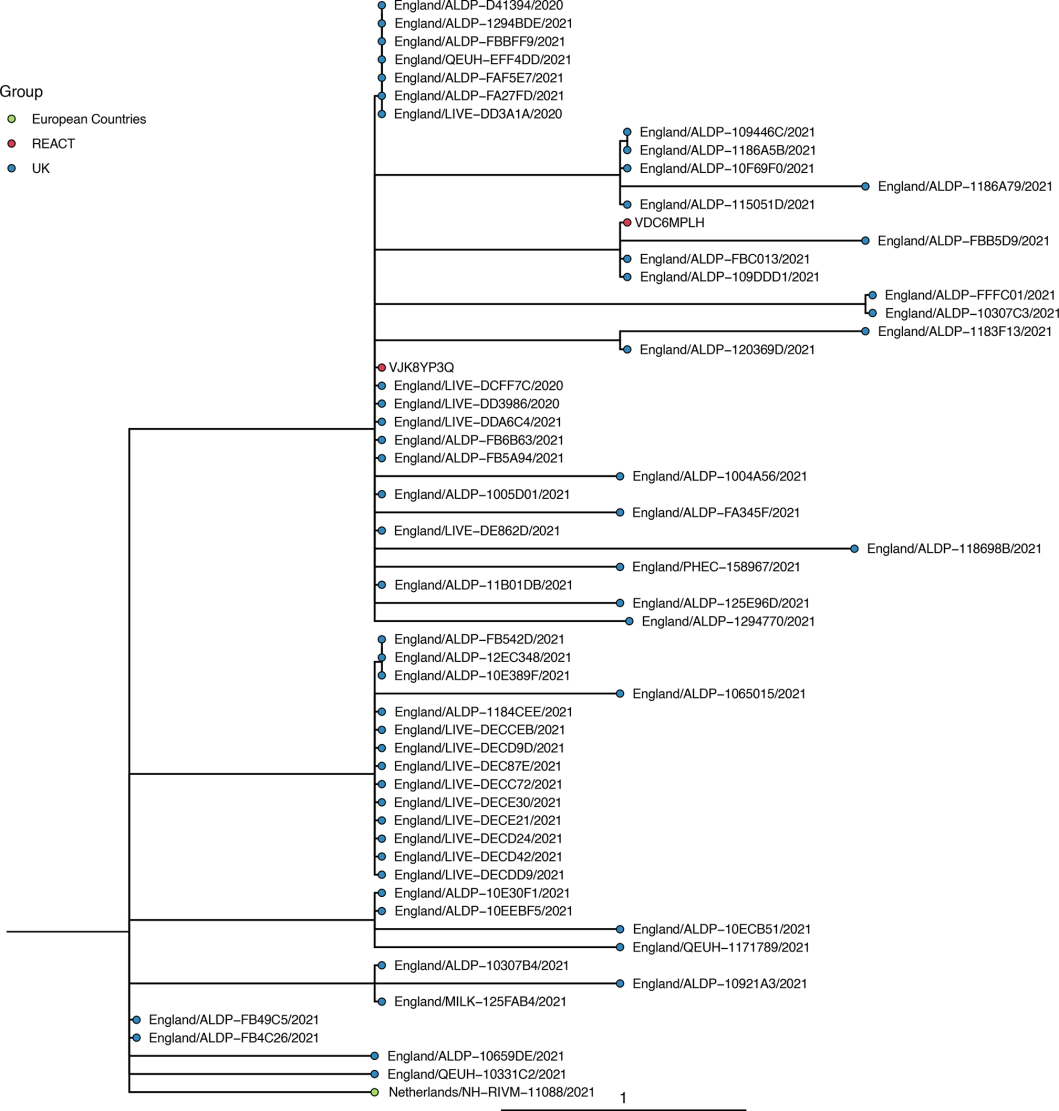
Phylogenetic tree showing the relation of A.23.1 lineages detected in REACT-1 to other A.23.1 sequences in the COG-UK database. REACT-1 sequences are coloured in red and have an ID beginning with the sequence ‘ARCH-’ next to them. All other sequences are coloured by the location in which the sequence was isolated.

**Fig. 6.**
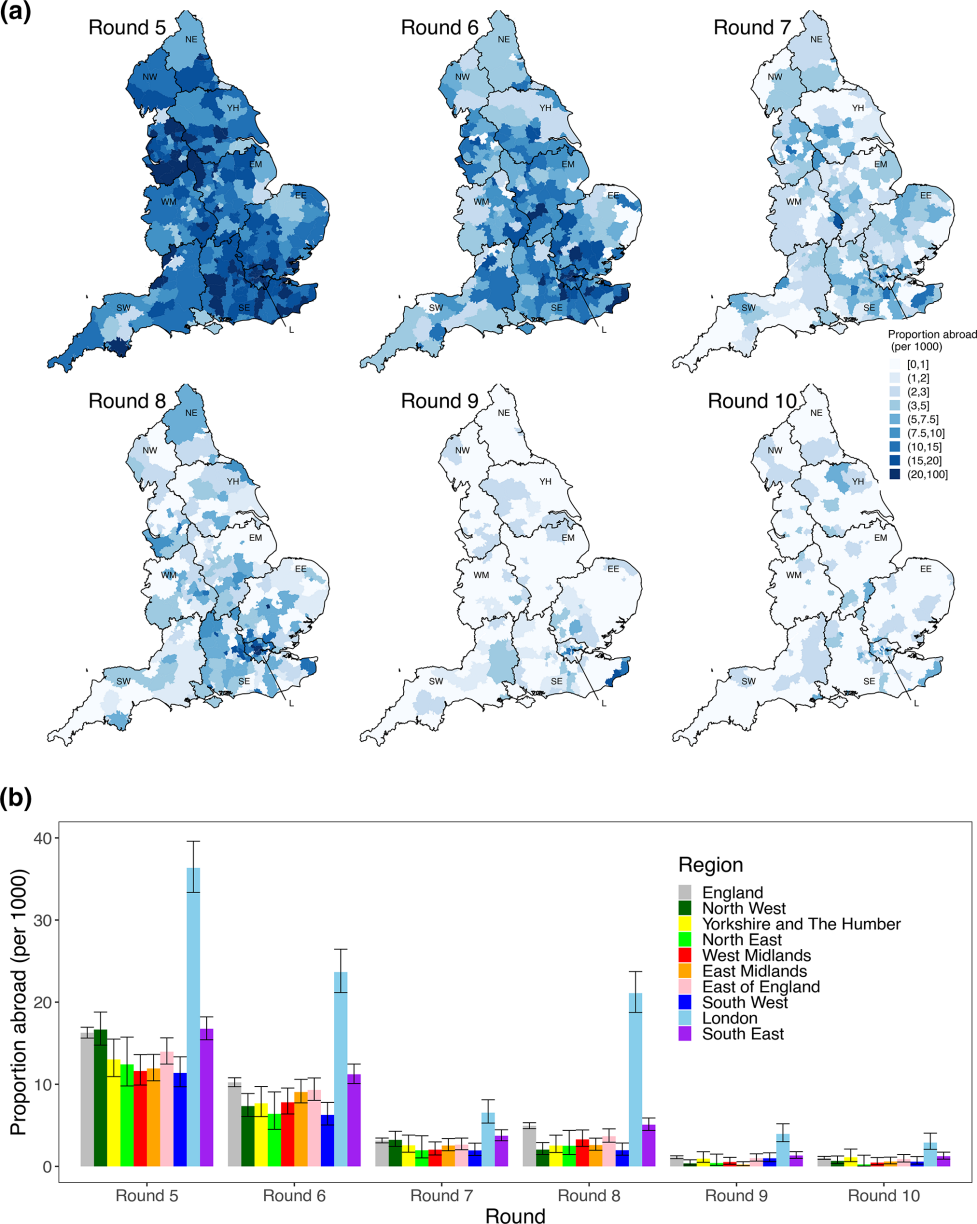
Patterns of frequency of returning from abroad in the prior 2 weeks. (a) Proportion of participants who answered they had been abroad in the previous 2 weeks by lower tier local authority. Regions: NE = North East, NW = North West, YH = Yorkshire and The Humber, EM = East Midlands, WM = West Midlands, EE = East of England, L = London, SE = South East, SW = South West. (b) Proportion of individuals who answered that they had been abroad in the previous 2 weeks by region and round. Dates: round 5=18 September–5 October 2020, round 6=16 October–2 November 2020, round 7=13 November–3 December 2020, round 8=6 January–22 January 2021, round 9=4 February–23 February 2021, round 10=11 March–30 March 2021.

## Methods

Methods for the REACT-1 study have been described previously [26]. Since May 2020, there have been 10 rounds of data collection approximately every month with between 140 000 and 175 000 swab tests completed over a 2–3 week period by a random subset of the population of England aged 5 and over. From round 8 onwards, all positive tests with a low N-gene Ct value (less than 34 used initially, but criteria changed midway through round 9 to less than 32 due to high rate of sequencing failure in those with N-gene Ct >32 [approx 88 %]) and a high enough volume were sent for genome sequencing (Public Health England Research Ethics Governance Group [reference: R and D NR0195]). Extracted RNA was amplified using the ARTIC protocol [27] with sequencing libraries prepared using CoronaHiT [28], and sequenced on the Illumina NextSeq 500 platform. Each set of 96 samples included one positive and one negative control. Raw sequencing data were analysed using the ARTIC bioinformatic pipeline [29] and uploaded to CLIMB [30] for further analysis.

From the genome sequences lineages are assigned using a machine-learning-based assignment algorithm, PangoLEARN [31] (database version 28 April 2021) with lineage assignment following the Pangolin nomenclature [9]. Not all obtained sequences were of a high enough quality for a lineage to be determined and so were not included in the analysis. Further, samples in which less than 50% of bases were covered were excluded from the analysis. A diagram showing how many positive samples were sequenced and how many had a lineage determined is shown in Fig. S1.

Following lineage allocation by the algorithm, each sequence was then also investigated individually, particularly for the presence of lineage-defining SNPs. This allowed for lineages that were too low quality to be called by PangoLEARN to be manually assigned. This occurred twice, once for a B.1 lineage call that exhibited 6 of 14 B.1.351 (Beta) lineage defining mutations (adjusted to Beta), and once for a B.1 lineage call that exhibited 11 out of 16 B.1.525 (Eta) lineage defining mutations (adjusted to Eta). In-depth analysis of low coverage regions of both samples, which fell below the normal minimum threshold of 10× coverage showed that all lineage defining mutations were present in at least one sequence read, further supporting these probable lineage designations. The thresholds for ‘probable’ lineage designations are defined by Public Health England [32]. Alpha lineages that also had the E484K SNP present were designated as a separate lineage (Alpha +E484K).

Phylogenetic trees were constructed in order to investigate how detected VOCs and VUIs fit into the wider epidemic context. A subsample of sequences for each variant was selected from a curated database of COG-UK up to 12 April 2021. A representative subsample for each lineage was selected using Civet [33] with a collapse threshold of 2. In total, 212 of 1583 Beta sequences and 21 of 60 A.23.1 sequences were selected. For each variant the maximum-likelihood phylogenetic tree was constructed using a HKY model implemented using IQ-TREE [34].

Then, 95% confidence intervals in lineage proportions were calculated assuming a binomial distribution using the Wilson method [35], which is preferred when the number of positive outcomes is small [36]. Differences in multinomial proportions between rounds were assessed using a multinomial goodness-of-fit test implemented using the XNomial package in R [37].

Potential confounding effects of region and age group on Alpha proportion during round 8 were investigated using logistic regression with a binomial likelihood and logit link function. Lineage assignment of Alpha versus any other was the binary outcome variable and both region and age group were included as covariates. Similar analysis was not attempted on round 9 and round 10 due to the small number of non-Alpha lineages.

Estimates of the average true number of swab positive cases by lineage at any one time during rounds 8, 9 and 10 were calculated by multiplying the estimates of weighted prevalence for rounds 8, 9 and 10 [12], the proportion of each lineage for rounds 8, 9 and 10 (Tables S1 and S2), and the population size of England and each region [38].

Relative differences in growth rates between two lineages were estimated by fitting a Bayesian logistic regression model to the binary lineage outcome. This was converted into an additive difference ( Δ ) in R through the equation $\Delta R\;=\;\Delta r\;\times \underline{g}$ , with the assumption $R=1\;+r\times \underline{g}$ [39] where *r* is the growth rate of a lineage and $\underline{g}$ is the mean generation time, assumed to be 6.29 days [40] for both lineages.

Smoothed spatial estimates of the relative proportion of two lineages were estimated using a Bayesian generalised-linear mixed-effects model implemented in the R package glmmfields [41]. We included 25 knots to describe the spatial processes and random spatial effects were assumed to follow a multivariate-*t* distribution. Priors of the model were chosen to be uninformative.

A subsample of positive participants in round 8 underwent a lateral flow immunoassay [42] approximately 6 weeks after their initial swab test. Differences in raw antibody positivity by lineages were assessed using logistic regression, with a binomial likelihood using a logit link function, and the result of the antibody test (positive/negative) as the binary outcome variable. Regression was performed using the subset of the data in which both the N-gene and E-gene had been detected. Further regression models were performed including different combinations of age, N-gene Ct value and E-gene Ct value as additional covariates. Further exploratory analyses were conducted with models including interaction terms between different combinations of variables and smoothed terms for some variables (not reported).

## Results

### Lineage diversity

In round 8 we were able to reliably determine lineages for 1088 out of 2282 positive samples, of which 83% (80 %, 85 %, *n*=898) were the Alpha variant, 0.37% (0.14 %, 0.94 %, *n*=4) were the Beta variant, 0.18% (0.05 %, 0.67 %, *n*=2) were the A.23.1 lineage, 0.09% (0.02 %, 0.52 %, *n*=1) were the Eta variant, and 0.18% (0.05 %, 0.67 %, *n*=2) were Alpha with the E484K SNP (Alpha +E484K, first identified in a cluster of cases in Bristol, UK) (Table S1, available in the online version of this article). The remaining 17% (15 %, 19 %, *n*=181) of lineages were classified as wild-type and comprised 35 distinct lineages, the main constituent of which was B.1.177 (*n*=105). In round 9, 236 lineages out of 689 positives were determined, of which 96% (92 %, 98 %, *n*=226) were the Alpha variant and the remaining 4.2% (2.3 %, 7.6 %, *n*=10) were classified as wild-type. In round 10, all 73 lineages, determined from 227 positive samples, were Alpha. Despite the reduced number of samples in round 10, we detect a significant decrease in diversity over all three rounds (*P*<0.001 for reduction in the proportion not Alpha). Over these three rounds national prevalence and infection incidence in England decreased sharply [43].

### Quantifying transmissibility of Alpha

Fitting a logistic regression model to whether a sample was identified as Alpha or not allowed us to estimate a difference in daily growth rate between Alpha and all other lineages of 0.049 (0.034, 0.067) (Fig. 1). This corresponds to an additive R advantage of 0.31 (0.21, 0.42) (assuming a mean generation time of 6.29 days, see Methods).

Proportions of Alpha showed marked spatial heterogeneity in January (round 8), with regions in the Midlands and the North of England showing lower proportions of Alpha compared to regions in the South (Fig. 1, Table S2). Sub-regional analysis showed a similar trend with a smoothed term regression model (see Methods) showing lower proportions of Alpha in areas of the Midlands, Yorkshire and The Humber, and the North West (Fig. 2). By February (round 9), spatial heterogeneity was substantially reduced with Alpha accounting for over 80% of lineages in all regions.

During round 8 there were higher proportions of Alpha in 18 to 54 year olds compared with other age groups (Fig. 1). Using a logistic regression model (see Methods), this pattern was not explained by regional confounding (Fig. 1). In contrast, for round 9, albeit based on fewer positive samples, the proportion of Alpha was similar in all age groups.

### Rates of symptom reporting

The percentage of people infected with Alpha reporting no symptoms (Fig. 3, Table S3) in the month prior to providing a swab was 33.3% (30.4 %, 36.3 %), compared with 38.2% (30.8 %, 46.1 %) for wild-type (*P*=0.24). Looking at the percentage of people reporting COVID-19 like symptoms (loss or change of sense of taste, loss or change of sense of smell, new persistent cough, fever) in the last week we found similar percentages exhibiting these symptoms between lineages with 45.7% (42.6 %, 48.8 %) for Alpha, compared with 45.4% (37.7 %, 53.3 %) for the wild-type (*P*=0.94).

### Differences in cycle threshold values

Quantitative PCR Ct values for N- and E-gene targets were lower for the Alpha variant relative to the wild-type lineages (Table 1, Fig. S2). Mean N-gene Ct value was 1.33 (0.60, 2.06) lower (*P*<0.001) and mean E-gene Ct value was 0.90 (0.14, 1.67) lower (*P*=0.020). These values are indicative of a higher viral load (Ct values are an approximate proxy to viral load) in those infected with Alpha with a decrease in Ct of 1 corresponding to an approximate twofold increase in viral load [44].

### Differences in antibody positivity

Antibody positivity 6 weeks after the initial swab test, assessed using a lateral flow immunoassay [42 ], was higher in those infected with the Alpha variant relative to those infected with wild-type lineages (Fig. 3, Table S4). Antibody positivity was 83.4% (79.7 %, 86.5 %) in those previously infected with Alpha and 72.8% (63.0 %, 80.9 %) in those previously infected with wild-type lineages (*P*=0.018). This difference was not explained by patterns in N-gene Ct value, E-gene Ct value, or age (Fig. 3). For example, the odds of Alpha-positive participants being sero-positive were 1.84 (1.08, 3.13) higher than those who were wild-type-positive, using multivariable logistic regression that included N-gene Ct value and age as covariates. (Model 6, Table S5.)

### Variants of concern and variants under investigation

Though only a small number of Beta, Eta, A.23.1 and Alpha +E484K were detected in January, because of the random sampling strategy used for REACT-1, we can estimate their prevalence with well-quantified uncertainty (Table S6). The single detection of any lineage in round 8 corresponded to an estimated 812 (136, 4847) swab positive infections in England at any one time (Table S6), suggesting that these lineages were already established in the community during January 2021. Additionally, none of the individuals infected with these lineages who answered the question reported that they had been abroad in the previous 2 weeks (Table S7). However, not all of the participants who tested positive for a VOC or VUI answered the question about recent travel (one did not for Eta, one did not for A.23.1). Also, the sequences of some of the REACT-1 samples grouped very closely with other English isolates when compared to a representative global subsample of the lineage (e.g. ARCH-000045H2 for Beta in Fig. 4 and all isolates in Fig. 5), further suggesting that significant local transmission was occurring at that time.

Geographically, both samples of A.23.1 were detected in the North West (Fig. 2, Table S2). Alpha +E484K was detected in London and the North West. The four Beta samples were detected in London (1), South East (1), and East of England (2), and the single Eta was also detected in London (Fig. 2).

None of these VOCs or VOIs were detected in rounds 9 and 10 suggesting a decrease in their relative proportions. Fitting a logistic regression model to whether a sample was a specific VOC, VUI or Alpha, there was no evidence for a difference in transmissibility between A.23.1, Eta, Alpha +E484K and Alpha. However, for Beta, which had the most samples available (*n*=4), the growth rate was estimated to be 0.110 (0.339, 0.002) less than Alpha (*P*=0.02) (Table S8). The converse is seen in publicly available sequence data from community testing [45] (Fig. S3), which, for the same period of time, found that the frequency of Beta grew faster than Alpha (Table S8, Fig. S4).

### Self-reported history of recent travel

Spatial patterns of observed VOCs and VOIs may be driven partly by geographical variation in the frequency with which people travel abroad. The overall proportion of participants reporting travel abroad in the past 2 weeks (Fig. 6, Table S9) in round 5 (September) was 1.63% (1.56 %, 1.69 %), but declined to 0.49% (0.46 %, 0.53 %) in round 8 (January), 0.11% (0.09 %, 0.13 %) in round 9 (February), and 0.10% (0.08 %, 0.12 %) in round 10 (March). London had the highest proportion and the South East the second highest for all rounds (Fig. 6). We estimated that over 55% of the people returning from abroad to England during rounds 8, 9 and 10 were in London and the South East (Table S9). Sub-regionally (Figs 6 and S5) we see that during round 5 (September) there was little spatial heterogeneity in the proportion of people who had been abroad 2 weeks prior, with similar proportions all across England. In contrast, during rounds 8, 9 and 10 there were high levels of heterogeneity with relatively higher proportions of travel among those living in central London and areas of Kent.

## Discussion

We describe lineage dynamics for SARS-CoV-2 in England for the period January to March 2021, based on representative community samples. From January to March 2021, the Alpha variant dominated the pandemic in England, further increasing in proportion over this time, spreading rapidly northwards and westwards. The estimated additive R advantage of 0.31 was smaller than estimates based on sequences collected from November 2021 to January 2021 in England (between 0.5 and 0.7) [8]. Our lower estimate of the difference in R at a later time is consistent with a decreasing selection coefficient reported in the earlier study. The increased transmissibility of Alpha over previously circulating lineages may be explained by higher viral loads; mean N- and E-gene Ct value was approximately 1 lower for Alpha relative to wild-type lineages. This result matches other work that suggested the Alpha variant has higher viral loads than previously circulating lineages [46]. However, it is possible that both analyses have been influenced by the high growth rate of Alpha during its emergence relative to other lineages, which could lead to a difference in the observed distribution generated by differences in the average time since infection [47]. Additionally, Ct value is only an approximate proxy to viral load and has the potential to be biassed due to rt-PCR testing not distinguishing between replicative and transcriptional RNA [48]. Also, given that our sample workflow ensures that lower Ct values are more likely to receive a lineage designation, a lower intrinsic Ct value for Alpha could have led to an overestimated proportion of Alpha in the community.

During January lower proportions of Alpha were detected in the Midlands and North of England, which is consistent with prior observations of the Alpha variant emerging in the South East [10] leading to earlier seeding events, and hence greater proportions of Alpha in the South of England. Greater proportions of Alpha were also detected in 18 to 54 year olds during January. However, case data from November and December 2020 showed a higher proportion of Alpha in school-aged children than in other age groups [8]. During this period schools were open for face-to-face teaching for all children, unlike during January when school attendance was greatly limited [49, 50], suggesting school closures had differing effects on the transmissibility of Alpha relative to wild-type lineages in different age groups.

Despite an increase in mortality observed for Alpha in other studies [51, 52] we find no evidence of a difference in the rate that infected individuals report the four classic COVID-19 symptoms (one or more of loss or change of sense of taste, loss or change of sense of smell, new persistent cough, fever) between Alpha and wild-type lineages. Our results describe only the lower part of the severity pyramid – the fraction of those infected who develop symptoms – and contrast with a previous study using clinical cases as the denominator, which found that the Alpha variant caused more severe illness with increased relative mortality [51, 52]. An ecological study has also found no difference in the symptomatology of Alpha against other previously circulating wild-type lineages [53]. We also note that participants in REACT-1 were not followed up, other than a small subset in round 8. Therefore, some participants will have developed symptoms after filling out the questionnaire. Though no differences in symptoms were detected, Alpha infections were found to have an increased odds of testing positive for antibodies 6 weeks later. This difference may be caused by the immune response itself or by other unmeasured confounders such as the time from infection to swabbing.

Our results suggest other lineages were circulating in the community at lower levels at the beginning of the study period, which coincided with the start of the third lockdown in England. In January, small numbers of Beta, Eta, A.23.1 and Alpha +E484K were detected; although the numbers were small, due to the size and sampling method of the study, they likely indicate a substantial level of community transmission. The lineage A.23.1 was first detected in England in the North West [16]. Given both community samples of A.23.1 in our dataset were also detected in the North West it suggests that the lineage continued to circulate locally with limited spread to other regions of England. In contrast, Alpha +E484K was detected in London and the North West, despite originally being detected in the South West [16], indicating either transmission out of the South West or that the E484K mutation arose independently within the Alpha variant. The Beta and Eta samples were all detected geographically close to London suggesting that the capital, a region with a far greater proportion of individuals travelling abroad, plays an important role in the importation of lineages.

These lineages were all then out-competed by Alpha during the course of the study. In the later rounds in February and March none of these lineages were detected — the decline in proportion of Beta relative to Alpha indicates that Beta was, on average, less transmissible than Alpha over this period. This period saw a sharp decline in SARS-CoV-2 infection incidence [43] and so it is possible that stochastic extinction of the far less prevalent Beta variant made it appear to be less transmissible than the highly prevalent Alpha variant; though other studies have also found evidence that Alpha is more transmissible than the Beta variant [54]. This may have been partly a result of targeted public health interventions and reductions in foreign travel. However, similar analysis performed on publicly available sequence data found that the frequency of Beta grew faster than Alpha over the same period of time. This difference likely reflects biases in public data, such as increased testing of international travellers and surge testing [55] in areas where variants are detected, demonstrating the importance of relatively unbiased sampling methods such as that used in REACT-1. The relative lineage dynamics do not seem to be consistent across space and time. Compared to patterns in England, higher proportions of Beta relative to Alpha have been seen in some regions of Europe [11, 14, 56] and in Africa [7].

Our study has limitations. During round 8 a subsample of participants testing positive also undertook two additional swab tests. The sequencing results from these additional swabs indicate possible misallocation of samples to participants. Though not all of these samples contained sufficient viral copies for successful sequencing (high Ct) or not enough physical volume in the sample, we were able to sequence multiple swab tests for some participants. These extra sequences, when a lineage was determined, allowed augmentation of the data for round 8 for some of the participants whose first test was unable to be sequenced or have a lineage designated. Thirteen of the 175 participants who had multiple tests had discordant lineage designations (Table S10). Four of these divergent lineage designations were not incongruent, for example B.1.351 (Beta) and B.1, likely reflecting a lower quality second sequence. For these four cases, the more advanced lineage was selected (B.1.351 over B.1). The remaining nine lineages could not be determined definitively, and so have been removed from the main analysis. This points to some potential sample mix-ups caused by manual cherry picking in the diagnostic pipeline; however, these errors are only likely to affect the most prevalent lineages, specifically Alpha and B.1.177.

Despite the relative success of Alpha against the prior wild-type and other circulating variants of concern during the first 3 months of 2021 in England, it too languished after the emergence of the far more transmissible Delta variant [12]. The immune landscape [57] against SARS-CoV-2 in the UK is rapidly changing due to a myriad of factors: natural infection, mass-vaccination, waning of immunity and further booster jabs. The potential for new variants to emerge with a fitness advantage due to an increase in transmissibility or the ability to evade existing patterns of immunity has been demonstrated by the recent rise of Omicron [58] and further variants remain a distinct possibility. The results of this paper demonstrate the importance of obtaining genomic sequence data on representative community samples, such as REACT-1, which is required for an unbiased analysis of changes in the epidemiology of the virus during periods of lineage competition.

## Data availability

### REACT-1 Data

Access to REACT-1 data is restricted due to ethical and security considerations. Summary statistics and descriptive tables from the current REACT-1 study are available in the Supplementary Material. Additional summary statistics and results from the REACT-1 programme are also available at https://www.imperial.ac.uk/medicine/research-and-impact/groups/react-study/real-time-assessment-of-community-transmission-findings/ and https://github.com/mrc-ide/reactidd/tree/master/inst/extdata REACT-1 Study Materials are available for each round at https://www.imperial.ac.uk/medicine/research-and-impact/groups/react-study/react-1-study-materials/

### Sequence data

Samples were deposited with appropriate public archives with individual accessions listed in ‘AccessionNumbers.xlsx’. Samples with >=50% coverage were deposited with the European Nucleotide Archive (https://www.ebi.ac.uk/ena/browser/view/PRJEB37886) for public use 15 without restriction, with consensus genomes given ERZ accession numbers and filtered sequence reads given ERR accession numbers. Additionally some consensus genomes were deposited with GISAID (https://www.gisaid.org), which imposes restrictions on reuse, and stricter quality control criteria are applied including a higher minimum coverage (>=90%). These arbitrary thresholds have no scientific basis and are under review, and if lowered will result in additional data being automatically released. Additional data is available from COG-UK (https://data.covid19.climb.ac.uk/) including multi-FASTA alignments, spreadsheets of metadata, up-to-date accessions and phylogenetic trees.

## Supplementary Data

Supplementary material 1

Supplementary material 2

Supplementary material 3

Supplementary material 4
