# Supplementary material for: The use of representative community samples to assess SARS-CoV-2 lineage competition: Alpha outcompetes Beta and wild-type in England from January to March 2021
Source: Microb Genom. 2023 Feb 6;9(2):mgen000887. doi: 10.1099/mgen.0.000887 (PMC9997751; doi:10.1099/mgen.0.000887)
Supplement: Supplementary material 1 [file mgen-9-887-s006.pdf]

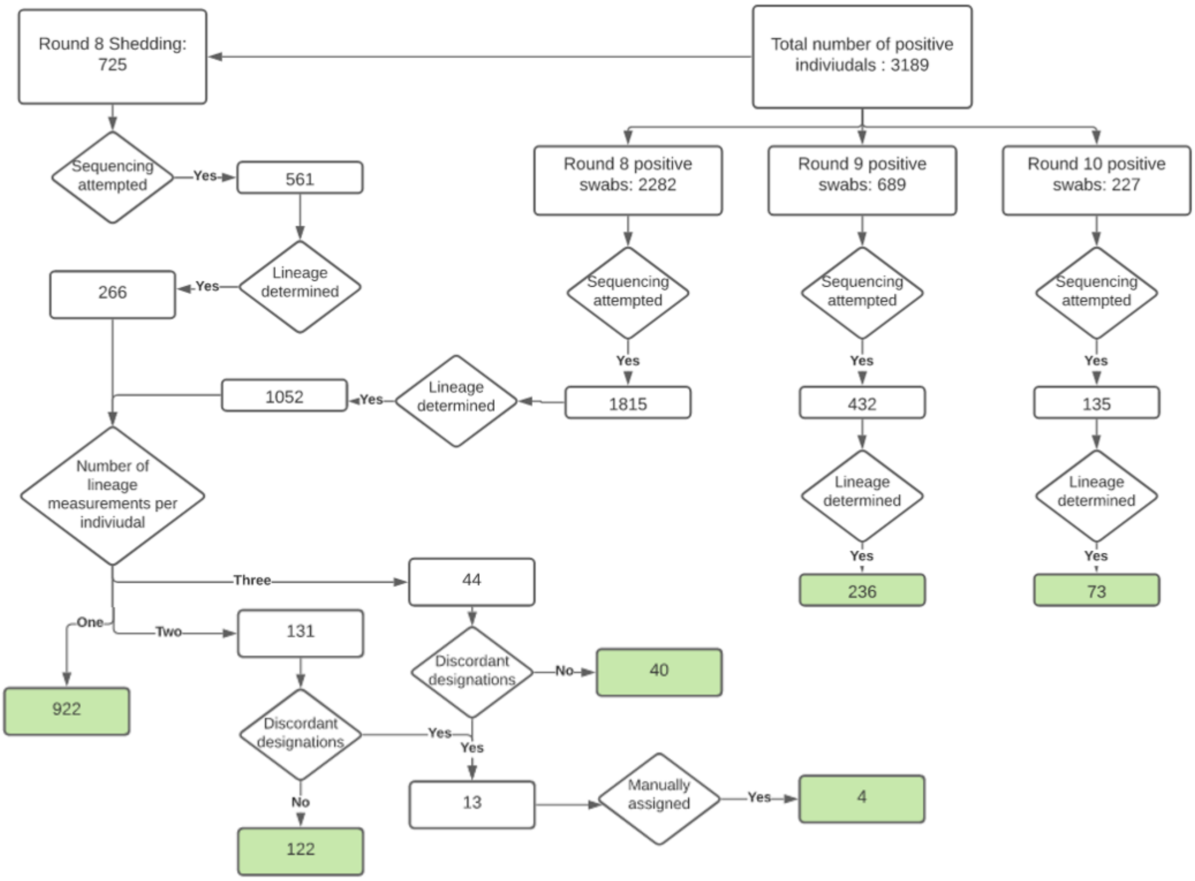

Supplementary Figure 1. Diagram showing the number of positive samples, the number for which sequencing was attempted, and the number of lineages that were determined and therefore used in the analysis (Green) for rounds 8, 9 and 10 of the study.

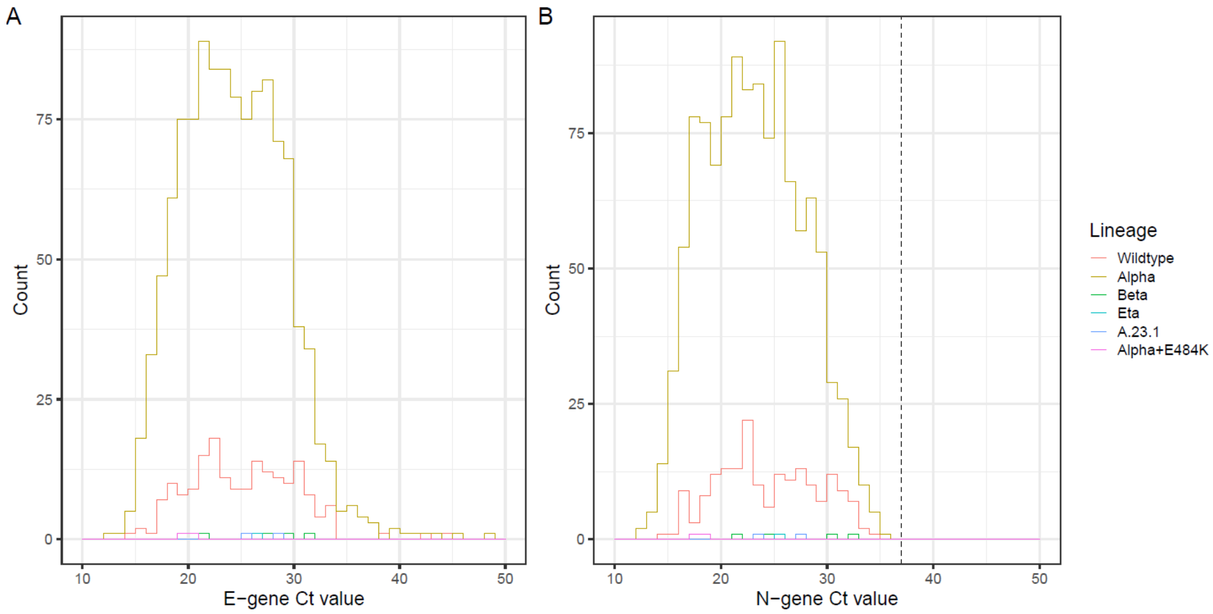

Supplementary Figure 2. Distribution of Ct values by lineage for (A) E-gene and (B) N-gene. Dotted line shows the N-gene Ct cutoff value of 37 that was used to define positivity.

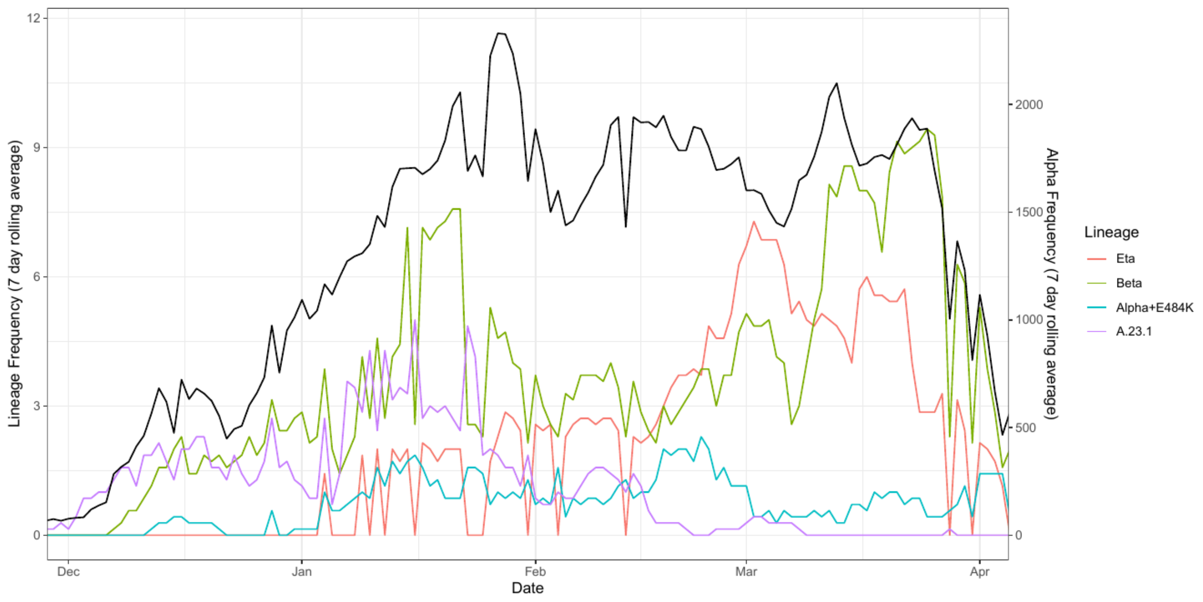

Supplementary Figure 3. Central 7 day rolling average of the frequency of Alpha (Black, right y-axis), Beta (Green, left y-axis), Eta (Red, left y-axis), A.23.1 (Purple, left y-axis) and Alpha+E484K (Blue, left y-axis) in publicly available data.

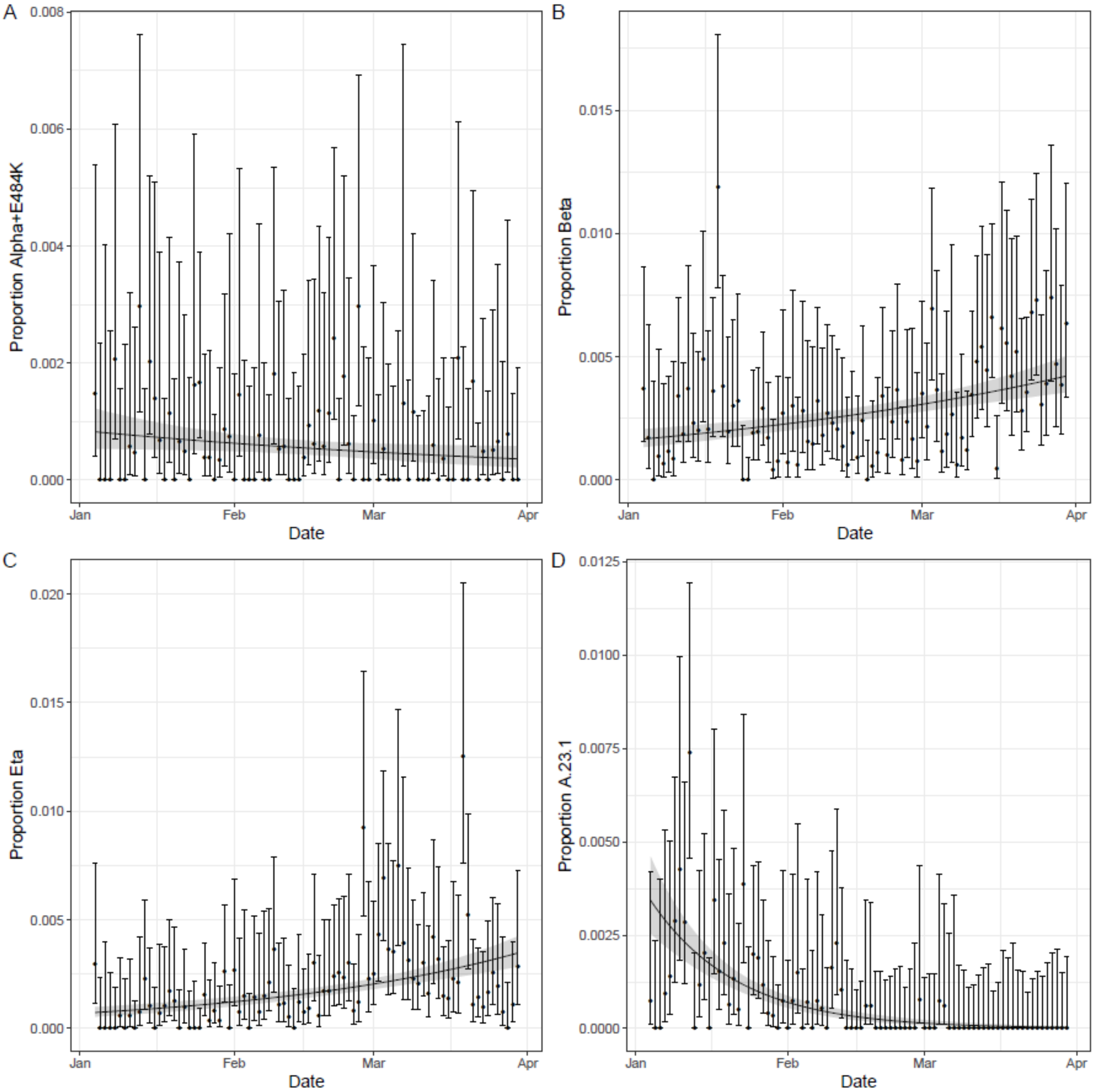

Supplementary Figure 4. Plot of the proportion of (A) Alpha+E484K (B) Beta (C) Eta and (D) A.23.1 relative to Alpha in publicly available data over the same time span as REACT-1 rounds 8 to 10 lineage data. Error bars show the 95% confidence interval for each daily proportion calculation. Shaded region shows the best fit Bayesian logistic regression model with 95% credible interval.

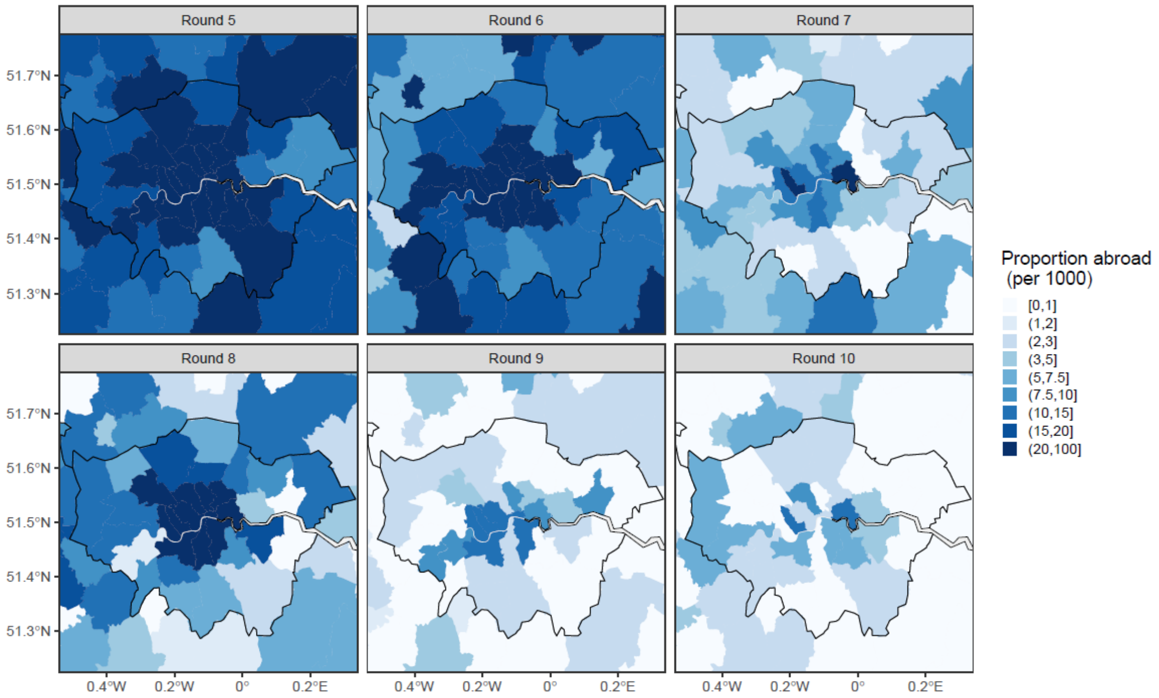

Supplementary Figure 5. Proportion of participants who answered they had been abroad in the previous two weeks by lower tier local authority zoomed into the London region. Dates: Round 5 =18 September - 5 October 2020, Round 6 =16 October - 2 November 2020, Round 7 = 13 November - 3 December 2020, Round 8 = 6 January - 22 January 2021, Round 9 = 4 February - 23 February 2021, Round 10 = 11 March - 30 March 2021.

Supplementary Tables

All supplementary tables are available in the additional file ‘SupplementaryTables.xlsx’

Supplementary Table 1. Lineages detected in round 8, round 9 and round 10 of REACT-1.

Supplementary Table 2. Regional distribution of positives for lineages in England for round 8, 9 and 10.

Supplementary Table 3. Symptom status by lineage

Supplementary Table 4. Antibody positivity 6 weeks after a positive swab test by lineage type.

Supplementary Table 5. Multivariate logistic regression models to determine the effect of lineage on antibody positivity.

Supplementary Table 6. Estimates of the average true number of swab positive cases by lineage at any one time during round 8, 9 and 10 in England and in each region of England.

Supplementary Table 7. Number of participants self-reporting being abroad in the two weeks prior to taking their swab test by round and lineage type.

Supplementary Table 8. Implied difference in growth rates of detected VOCs and VUIs.

Supplementary Table 9. Proportion of participants that reported being abroad two weeks prior to their swab test by region and by round, estimated average number of people who had been abroad in the previous two weeks by region and round, and the overall percentage of people returning from abroad that were in each region of England.

Supplementary Table 10. Description of the participants who had discordant lineage designations and the overall lineage designation.
